# Dry Needling of Anterior Neck Muscles in a Patient With Muscle Tension Dysphonia: A Case Report

**DOI:** 10.1002/ccr3.70832

**Published:** 2025-09-08

**Authors:** Yazan Alkhatib

**Affiliations:** ^1^ Myopain Medical Center Doha Qatar

**Keywords:** dry needling, muscle tension dysphonia, myofascial trigger points, physiotherapy, voice disorders, voice therapy

## Abstract

Muscle tension dysphonia (MTD) is a functional voice disorder commonly treated with voice therapy. However, the role of physiotherapy—specifically dry needling (DN)—has not been explored in the literature. This case report describes a 40‐year‐old male with persistent vocal fatigue, hoarseness, and anterior neck tightness associated with MTD. He underwent three sessions of DN targeting the anterior neck muscles. Outcome measures included the Voice Handicap Index (VHI), which improved from 73 to 54, and the Voice‐Related Quality of Life (V‐RQOL) score, which improved from 37 to 24. The patient also reported improved voice clarity, reduced vocal effort, and decreased neck discomfort. This case suggests DN may be a promising adjunctive intervention for MTD and highlights the need for further investigation into its role in interdisciplinary voice care.

**Summary**

- Dry needling of anterior neck muscles may reduce vocal strain and improve voice quality in patients with muscle tension dysphonia.
- This case highlights a novel, physiotherapy‐based intervention that may complement traditional voice therapy in managing voice‐related muscular tension.

## 1 Introduction

Muscle tension dysphonia (MTD) is a functional voice disorder characterized by excessive and dysregulated muscular activity in the laryngeal and extralaryngeal muscles, leading to vocal impairment in the absence of structural abnormalities [1]. It is commonly associated with vocal fatigue, strain, hoarseness, and discomfort [2]. Management of MTD traditionally involves voice therapy provided by speech‐language pathologists, targeting vocal habits, breath support, and muscle tension reduction. However, there is limited literature addressing physical therapy interventions—particularly dry needling—for MTD.

Dry needling (DN) is a minimally invasive technique used by physiotherapists to treat myofascial trigger points and associated muscle tension. DN has been shown to be effective in managing various musculoskeletal conditions, particularly those related to chronic pain and muscular dysfunction, such as tension‐type headaches and cervicogenic headaches [3]. These conditions share a common pathophysiological feature with MTD: overactivation of specific muscle groups.

To date, there are no published studies exploring the use of DN as a treatment for patients with MTD. This case report presents the clinical outcome of a 40‐year‐old male patient diagnosed with MTD who underwent DN treatment of anterior neck muscles, aiming to evaluate its effect on vocal function using the voice handicap index (VHI) [4] and evalute the MTD quality of life aspects using the voice‐related quaility of life (V‐RQOL) [5]. The physiotherapist (author), with expertise in DN but unfamiliar with MTD at the time, documented this case to share clinical reasoning, treatment approach, and results with the wider physiotherapy community.

This case may represent a novel, adjunctive approach in the interdisciplinary management of MTD and could lay the groundwork for future research in this area.

## 2 Case History and Examination

A 40‐year‐old male patient presented directly to our physiotherapy clinic with complaints of persistent vocal fatigue, hoarseness, and a sensation of tightness in the front of the neck during speech. He had recently been diagnosed with MTD by a specialist, but he did not receive a referral for physiotherapy. Instead, the patient independently sought treatment after reading about the potential role of DN in reducing muscle tension. He was encouraged to try this approach by a friend who had experienced improvement in neck pain following DN.

This was the patient's first attempt to address his voice dysfunction through physical therapy. At the time of presentation, he had undergone voice therapy but not any manual therapy interventions for his condition. The patient reported that his symptoms were affecting his ability to speak for prolonged periods, communicate clearly, and participate in social and professional conversations.

During the physiotherapy assessment, palpable tuat band with hypersensitive spot and referred pain were identified in the anterior neck muscles, especially the sternocleidomastoid and anterior scalenes confirming the presence of myofascial trigger points [6]. These findings were consistent with the characteristic muscle overactivity seen in MTD. The patient had no contraindications to DN and provided informed consent for a trial of treatment.

## 3 Differential Diagnosis, Investigations and Treatment

The treatment plan consisted of three sessions of DN, delivered once per week over a period of three weeks. The intervention focused on reducing myofascial tension in the anterior neck musculature associated with phonation and vocal effort.

Targeted muscles included: sternocleidomastoid, anterior scalene, middle scalene, anterior digastric, posterior digastric, and longus colli.

These muscles were selected based on clinical palpation findings and anatomical guidelines from “Trigger Point Dry Needling: An Evidence and Clinical‐Based Approach” by Dr. Jan Dommerholt [7]. It is important to note that while some deeper anterior neck muscles—such as the omohyoid and sternohyoid—may also contribute to excessive tension, they are not included in Dr. Dommerholt's dry needling protocol due to their proximity to critical vascular structures such as carotid artery and internal jugular vein, making them unsafe to needle.

All needling procedures were performed under aseptic conditions. The patient was placed in a supine position. Each session was followed by post‐needling care instructions, and no significant adverse effects were reported.

Outcome measures used to assess progress included:

Voice Handicap Index (VHI): Decreased from 73 (severe handicap) to 54 (moderate handicap).

Voice‐Related Quality of Life (V‐RQOL): Improved from 37 (fair) to 24, reflecting a meaningful enhancement in voice‐related daily functioning.

In addition to the quantitative outcomes, the patient reported substantial subjective improvements in his vocal ability. He described feeling less strain and effort while speaking, as well as greater ease and confidence during communication, particularly in social and professional settings. The tightness and discomfort he previously felt in the anterior neck also noticeably decreased.

The patient expressed high satisfaction with the intervention. The results were unexpected for both the clinician and the patient, especially given the absence of existing literature supporting DN as a treatment for MTD. These findings prompted the decision to document and share the case with the broader physiotherapy and voice care community (Tables 1 and 2).

**TABLE 1:** Voice Handicap Index (VHI) [4] scores before and after dry needling.

Domain	Before	After	Improvement	% Change
Functional	29	20	9	31%
Physical	22	20	2	9.1%
Emotional	22	14	8	36.4%
Total	73	54	19	26%

**TABLE 2:** Voice‐Related Quality of Life (V‐RQOL) [5] scores before and after dry needling.

Domain	Before	After	Improvement	% Change
Physical functioning	17	10	7	41.2%
Social–emotional	20	14	6	30.0%
Total	37	24	13	35.1%

## 4 Conclusion and Results (Outcome and Follow‐Up)

This case report highlights a positive clinical outcome following DN of anterior neck muscles in a patient with MTD, demonstrating improvement in voice quality and patient‐reported outcomes.

The observed improvements in VHI (73 to 54) and V‐RQOL (37 to 24) scores are clinically significant. They suggest that DN may contribute to decreased muscular tension affecting voice function and overall quality of life in individuals with MTD. Additionally, the patient's subjective reports of vocal ease and reduced neck tightness reinforce the potential of DN as a supportive treatment strategy.

This case report highlights the potential role of DN as a novel, adjunctive intervention for managing MTD. Following three sessions of DN targeting anterior neck muscles, the patient experienced clinically meaningful improvements in both voice‐related disability and quality of life, as measured by the VHI and the V‐RQOL scales.

To the best of our knowledge, this is the first documented case exploring DN in the treatment of MTD. The positive outcomes observed suggest that reducing muscle tension in the cervical region may play an important role in alleviating symptoms in select patients.

Given the absence of current evidence and clinical guidelines on this approach, further investigation through well‐designed clinical trials is needed to validate these preliminary findings. ENT specialists and allied health professionals should be informed of DN's potential utility, and consider collaborative, interdisciplinary strategies when managing complex cases of voice dysfunction related to muscle tension.

## 5 Discussion

This case report documents the novel use of DN as an adjunctive intervention for MTD, with clinically meaningful improvements in both subjective and objective outcomes following three sessions of targeted DN to anterior neck muscles.

MTD is a functional voice disorder typically managed through behavioral voice therapy [8]. It is characterized by excessive muscular activity in the laryngeal and extralaryngeal musculature [2], which may persist even after voice therapy. While the underlying mechanisms are not yet fully understood, MTD is known to involve muscular hyperfunction and maladaptive compensatory patterns [2]. The contribution of neck and upper thoracic musculature to voice production—and their potential overuse—has been acknowledged in both speech‐language pathology [9] and physiotherapy literature, but direct physical therapy interventions remain underexplored [10, 11].

Dry needling is increasingly used by physiotherapists to address myofascial trigger points and reduce muscle tension in musculoskeletal conditions, such as tension‐type headaches and cervicogenic headaches [3]. These conditions, like MTD, involve muscular overactivation and pain referral patterns originating from taut bands in the cervical muscles. In this case, the physiotherapist recognized a parallel between muscle‐related voice dysfunction and other tension‐based syndromes, prompting the decision to apply DN to the anterior neck muscles commonly implicated in both voice production and neck dysfunction.

Recent literature has begun to explore the role of physical therapy in the management of muscle tension dysphonia (MTD), with encouraging but varied findings. Tate et al. [11] conducted a large retrospective cohort study (*n* = 178) and demonstrated that manual physical therapy, including myofascial release, produced significant improvements in VHI‐10 scores for patients with cervicalgia, with mean gains of 9.95 points when combined with voice therapy and 8.31 points with physical therapy alone, suggesting that targeted physiotherapy can benefit patients even in the absence of traditional voice therapy. Similarly, Craig et al. [10], in a cohort of 153 patients, reported that voice therapy alone yielded a significant improvement in VHI scores (~10‐point change), while physical therapy either alone or combined with voice therapy produced comparable but not statistically superior outcomes, highlighting that physiotherapy may provide adjunctive benefits but requires further validation. In contrast, Tomlinson and Archer [12] presented a case series illustrating that manual therapy combined with targeted exercises led to notable improvements in voice function, underscoring the potential for individualized physical therapy protocols to reduce vocal strain and improve functional outcomes. In line with these findings, the present case report adds novel evidence by demonstrating that dry needling of anterior neck muscles resulted in clinically meaningful improvements in both the Voice Handicap Index and Voice‐Related Quality of Life scores, suggesting that DN may represent a complementary physiotherapy‐based intervention for addressing cervical muscle overactivity in MTD. Collectively, these studies highlight the emerging role of physical therapy ranging from manual therapy and exercise to dry needling as a valuable adjunct in MTD management, while also emphasizing the need for randomized controlled trials to confirm efficacy and clarify its integration with conventional voice therapy.

Given these results, ENT specialists should be aware of DN as a potential adjunctive treatment option in cases of MTD—especially for patients with persistent muscle tension despite traditional voice therapy. Encouraging multidisciplinary collaboration between ENT specialists, speech‐language pathologists, and physiotherapists may enhance patient outcomes in complex cases of voice dysfunction.

This case appears to be the first published account exploring DN in a patient with MTD. While the findings are encouraging, several limitations must be acknowledged:

The report describes a single case without a control or comparison group.

Improvements may have been influenced by placebo effects or natural variation in symptoms.

Follow‐up was limited to the immediate posttreatment period, with no long‐term data.

Despite these limitations, the case raises important questions about the role of physiotherapy in the interdisciplinary management of voice disorders. It highlights the need for future research—particularly randomized controlled trials—to evaluate the safety, efficacy, and mechanisms of DN in patients with MTD.

## Author Contributions

**Yazan Alkhatib:** data curation, project administration, supervision, writing – original draft, writing – review and editing.

## Consent

Written informed consent was obtained from the patient for the publication of this case report and any accompanying clinical details. The patient reviewed the manuscript, confirmed the accuracy of the described treatment and outcomes, and agreed to the anonymous presentation of the case for scientific and educational purposes.

## Conflicts of Interest

The author declares no conflicts of interest.

## Data Availability

The data that support the findings of this study are available from the corresponding author upon reasonable request.
